# Appendicular agenesis as an unusual intraoperative finding in acute abdomen: Case report and literature review

**DOI:** 10.1016/j.ijscr.2021.106740

**Published:** 2021-12-29

**Authors:** Felipe Giron, Carlos Eduardo Rey Chaves, María Lina Rodríguez, Danny Conde, David Venegas, Alberto Arango

**Affiliations:** a Faculty of Medicine, Universidad del Rosario, Colombia; b Faculty of Medicine, Universidad de los Andes, Bogotá, Colombia; c Faculty of Medicine, Universidad del Bosque, Colombia; d Hospital Occidente de Kennedy, Colombia

**Keywords:** Appendicitis, Appendicular agenesis, Acute abdomen, Surgery

## Abstract

**Introduction:**

Appendicular agenesis is a rare condition that accounts for 1 of every 100,000 exploratory laparotomies, usually in clinical suspicion of appendiceal inflammatory processes with higher incidence in adults.

**Clinical findings:**

We present a case of a 19-year-old female with appendicular agenesis who underwent exploratory laparoscopy in the context of appendiceal inflammatory process.

**Conclusion:**

Vermiform appendix agenesis is a challenging diagnosis made in most cases as an intraoperatively finding. Systematic revision of abdominal cavity should be performed after ruling out other causes of acute abdomen. No implications have been described in patients with appendix abnormalities.

## Highlights

• Appendicular agenesis is a rare entity, there isn't clinical features that could increase the clinical suspicion.
• Intraoperative diagnosis is relatively frequent, and systematic revision of the cavity must be performed.
• Apparently, no clinical implications of appendicular agenesis are present.

## 1 Introduction and importance

First description of appendicular agenesis was made by Morgani et al. [1], incidence is approximately 1:100.000 laparotomies due to acute abdomen according to Chevre et al. [2], [3]. Non-specific causes of acute abdomen are usually due to acute appendicitis [2], and diagnosis of appendicular agenesis are frequently performed in an incidental way [4]. Abnormalities of the appendix are unusual, and frequently there are not found because they do not show any signs or symptoms [2], [3], [4].

.

## 2 Presentation of the case

After ethical and institutional approval, previous informed consent filled, following SCARE guidelines [5]. Our paper presents a 19-year-old woman, with no previous clinical history; who was admitted through the emergency room with 2 days of abdominal pain localized in the right lower quadrant (RLQ) of the abdomen, associated to nausea, and vomiting; Patient also presented anorexia, and bladder tenesmus. Patient had no history of gynecologic diseases.

Physical examination revealed tenderness in RLQ; associated with abdominal guarding. She had no fever or dyspnea. Gynecologic examination did not show signs that suggest pelvic inflammatory disease (No abnormal vaginal discharge, no pain or bleeding during intercourse and no pain during vaginal examination). White blood cell count reveals leukocytosis (17.370 μ/L), with neutrophilia (88%). Pregnancy tests were performed with negative results. Also, C reactive protein was analyzed and reports a negative value. Due to symptoms previously described, a urine test was ordered, with no signs of infection.

**Image 1:**
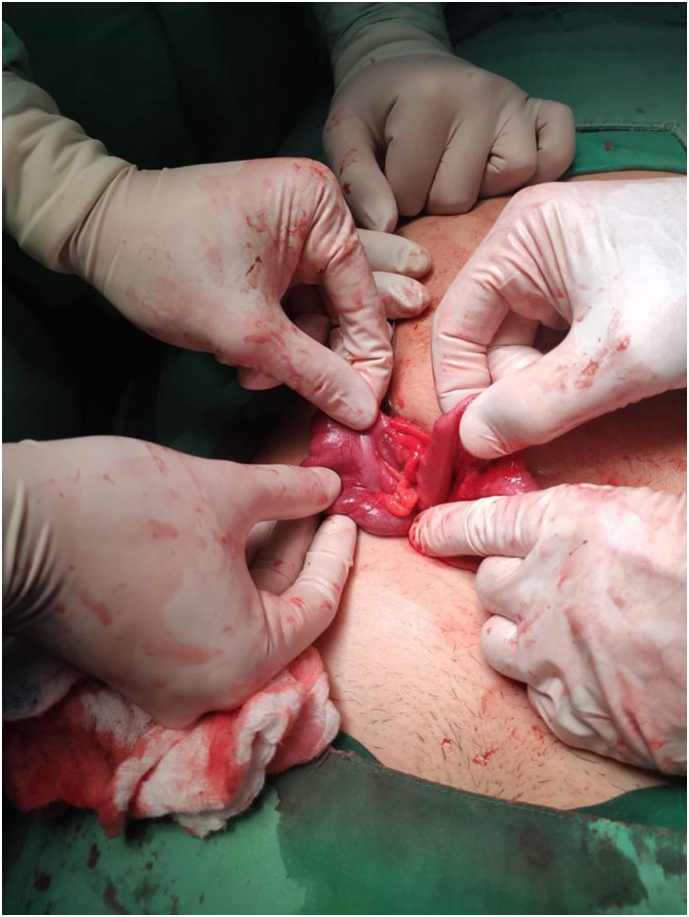
Intraoperative findings.

**Image 2:**
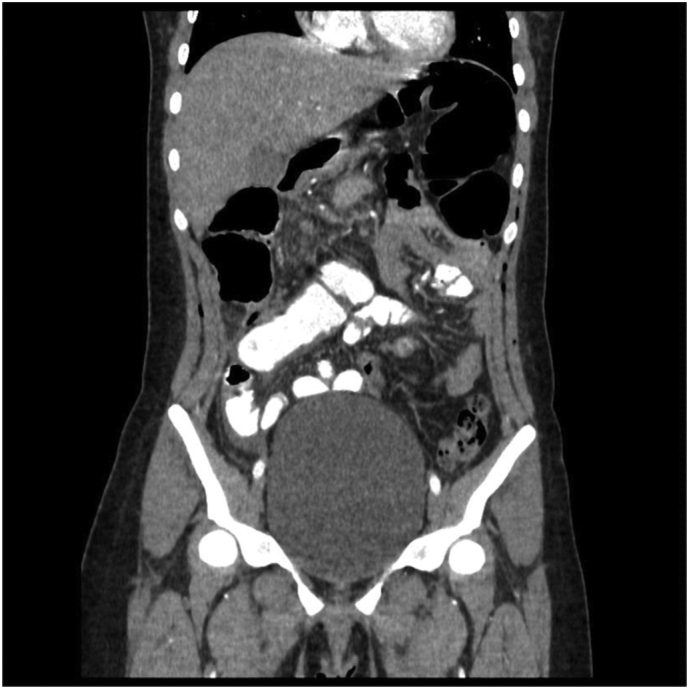
Tomographic findings.

Initial hydration and observation were performed. Appendicitis Inflammatory Response (AIR) Score was calculated (7 points). Due to intermediate risk, based on clinical findings, and worseness of clinical status and acute abdomen, surgical approach was considered with a suspected diagnosis of acute appendicitis. A Laparoscopic approach was performed. Told fascia was released on the ascending colon, identifiying the cecum and ilioceccum valve, following the taenias and its confluence we examined the entire colon, with no visualization or identification of the appendix. All surgical causes of acute abdomen were ruled out, pelvic cavity was revised, identifying signs of inflammatory pelvic disease. In furtherance of a proper revision a Rockey-Davis approach was performed, in order to exteriorize the cecum and ileum with a systematic review of the cavity. However, no appendix was found (Image 1). Contrasted computed abdominal tomography (CT) was performed in the postoperative period, with no visualization of the cecum appendix (Image 2); configuring the diagnosis of appendicular agenesia.

Surgical time was 120 min, no intraoperative bleeding was documented. In-hospital stay was 2 days. Gynecologic consultation was required in order to define treatment for inflammatory pelvic disease. Outpatient consultation after 2 weeks did not show any complication. No postoperative complications were documented after 30 days of follow up. His controls have been uneventful upto 3 months.

## 3 Discussion

Abnormalities of the vermiform appendix are rare, with an incidence that arounds 0.001% [6]. Its diagnosis is usually performed in an incidental way [6] being detected usually after an emergent surgical approach (Laparotomy in most cases) due to acute appendicitis suspicion. As a matter of fact, in some of the cases, appendix agenesia isn't found in live patients with an incidence in autopsies arounds 1:15.000 cases [6].

.In terms of embryology, vermiform appendix development starts at the 8th week [7], and is a tubular structure with a blind end that depends on the cecum; A fully developed structure is visible at the 10th week in gestational age [7], [8]. .Localization of the appendix is usually 2–5 cm below of the ileo-ceccum valve just in the junction of the three taenia. Some variations in its position have been described. Cilindro de Souza et al. [9], described a large series of cases that reports the retrocecal position of the vermiform appendix as the most frequent one (43.7%), followed by 24.4% of the cases in sub-cecal position [9].

.Embryological abnormalities of the vermiform appendix have been also documented. Collins in 1951 [10] described a full classification; Class I: Patient with failure of development of cecal anlage that traduce agenesis of both cecum and appendix (In major proportion in females, in the early childhood); Class II: Partial development of cecal anlage; rudimentary growth of the cecum, and no appendix (Founded in males in 70% of the cases, and in newborns); Class III: Full development of cecal anlage, and no development of appendiceal anlage; with normal growth of the cecum, and no appendix; founded in majority of the patients in adolescents and second decade of the life; Class IV: Fully development of cecal anlage, early cessation of appendiceal anlage, with normal development of the cecum and rudimentary appendix; and Class V: Fully development of the cecal anlage, and failure of the appendiceal anlage to become differentiated; however other abnormalities are described such as duplication, that could vary in 0.004% and described according to Cave-Wallbridge classification [11].

Failure in the appendix development according to Hei et al., could be attributed to an intrauterine vascular accident, and in some cases is associated with intestinal atresia [12], as well, toxic-associated with Thalidomide are described in literature as possible causes of appendix abnormalities [13]. Literature case reports of appendiceal absence are usually described in adult patients [1], [2], [3], [4], [5], [6], [7], [8], [9], [10]. There are no clinical manifestations of appendix agenesis, and usually are found in patients with acute abdomen suspicion that needs laparotomy, as in our case; following a systematic review of the cavity, no appendix were found. In our case, diagnosis was made intraoperative of a class III Collins appendix abnormality, and confirmed by a computed tomography.

## 4 Conclusion

Vermiform appendix agenesis diagnosis could be performed as an intraoperative finding, and systematic revision of the cavity should be done after rule out other causes of acute abdomen. Apparently, no implications were described in patients with appendix abnormalities.

## Provenance and peer review

Not commissioned, externally peer-reviewed.

## Consent

Written informed consent was obtained from the patient for publication of this case report and accompanying images. A copy of the written consent is available for review by the Editor-in-Chief of this journal on request.

## Ethical approval

Ethical approval of institutional committee was made previous publication.

## Funding information

This research did not receive any specific grant from funding agencies in the public, commercial, or not-for-profit sectors.

## Guarantor

Felipe Giron

## Research registration number

None.

## CRediT authorship contribution statement

**Felipe Giron, MD, MSc:** Make substantial contributions to conception and design, acquisition of data, analysis and interpretation of data**.**

**Carlos Rey, MD:** Participate in drafting the article and revising it critically for important intellectual content.

**Lina Marcela Rodriguez, MD:** Participate in drafting the article and revising it critically for important intellectual content.

**Danny Conde, MD:** Participate in drafting the article and revising it critically for important intellectual content.

**David Venegas, MD:** Participate in drafting the article and revising critically for important intellectual content.

**Alberto Arango, MD:** Give final approval of the version to be submitted and any revised version.

## Declaration of competing interest

Authors do not declare any conflict of interest.
